# Selective ansa cervicalis nerve transfer to the marginal mandibular nerve for lower lip reanimation: An anatomical study in cadavers and a case report

**DOI:** 10.1002/micr.30992

**Published:** 2022-12-13

**Authors:** Villiam Vejbrink Kildal, Richard Tee, Lukas Reissig, Wolfgang J. Weninger, Chieh‐Han John Tzou, Andrés Rodriguez‐Lorenzo

**Affiliations:** ^1^ Department of Plastic and Maxillofacial Surgery Uppsala University Hospital Uppsala Sweden; ^2^ Department of Surgical Sciences Uppsala University Uppsala Sweden; ^3^ Division of Anatomy Medical University of Vienna Vienna Austria; ^4^ BioImaging Austria (CMI) Vienna Austria; ^5^ Plastic and Reconstructive Surgery, Department of Surgery Hospital of Divine Savior (Krankenhaus Goettlicher Heiland) Vienna Austria; ^6^ Faculty of Medicine Sigmund Freud University Vienna Austria; ^7^ Facial Palsy Center TZOU Medical Vienna Austria

## Abstract

**Background:**

Donor nerve options for lower lip reanimation are limited in patients undergoing oncological resection of the facial nerve. The ansa cervicalis nerve (ACN) is an advantageously situated donor with great potential but has not been examined in detail. In the current study, the anatomical technical feasibility of selective ACN to marginal mandibular nerve (MMN) transfer for restoration of lower lip tone and symmetry was explored. A clinical case is presented.

**Methods:**

Dissections were conducted in 21 hemifaces in non‐embalmed human cadavers. The maximal harvestable length of ACN was measured and transfer to MMN was simulated. A 28‐year‐old male underwent ACN‐MMN transfer after parotidectomy (carcinoma) and was evaluated 12 months post‐operatively (modified Terzis' Lower Lip Grading Scale [25 observers] and photogrammetry).

**Results:**

The harvestable length of ACN was 100 ± 12 mm. A clinically significant anatomical variant (“short ansa”) was present in 33% of cases (length: 37 ± 12 mm). Tensionless coaptation was possible in all cases only when using a modification of the surgical technique in “short ansa” cases (using an infrahyoid muscle nerve branch as an extension). The post‐operative course of the clinical case was uneventful without complications, with improvement in tone, symmetry, and function at the lower lip at 12‐month post‐operative follow‐up.

**Conclusions:**

Selective ACN‐MMN nerve transfer is anatomically feasible in facial paralysis following oncological ablative procedures. It allows direct nerve coaptation without significant donor site morbidity. The clinical case showed good outcomes 12 months post‐operatively. A strategy when encountering the “short ansa” anatomical variant in clinical cases is proposed.

## 1 INTRODUCTION

Patients with facial nerve resection from ablative oncological procedures are traditionally regarded as poor candidates for facial nerve reanimation. This is often attributed to advanced age, presence of multiple comorbidities, adjuvant radiotherapy, and poor survival prognosis (Klein et al., 2019). However, recent studies have shown that neither age (Hembd et al., 2018) nor post‐operative radiation therapy (Gidley et al., 2010) preclude good and consistent reanimation outcomes.

Among the facial nerve branches, the marginal mandibular branch of the facial nerve (MMN) has often been neglected in facial reanimation (Mandlik et al., 2019; Terzis & Kalantarian, 2000), but has received increasing attention in recent years (Tzafetta et al., 2021). While several options for nerve reanimation exist in oncological patients (Bianchi et al., 2012), a regional nerve transfer procedure offers the benefits provided by a vascularized donor nerve in proximity to the recipient site and completion in a single stage. These attributes potentially confer improved nerve recovery in an irradiated field which, together with a shorter reinnervation time, can be critical in the intended patient demographic (Klein et al., 2019). Potential methods and donor nerves for lower lip reanimation include end‐to‐side hypoglossal transfer (Atlas & Lowinger, 1997; Coyle et al., 2013; Koh et al., 2002; Yetiser & Karapinar, 2007) and intra‐facial transfer with the cervical branch of the facial nerve (Rodriguez‐Lorenzo et al., 2016). However, in oncological facial nerve resection, the recipient facial nerve branch may not reach the hypoglossal nerve, while the cervical branch may reside within the tumor margin. To avoid the use of a nerve graft, an extra‐facial nerve transfer option with good reach is required in the area. This led the authors to examine the use of the ansa cervicalis nerve (ACN) as a regional donor.

The superior root of the ACN (formerly known as the descendens hypoglossi or ansa hypoglossi; Kikuta et al., 2019; Olry & Haines, 2002) is advantageously located in the neck and has been used for decades in otolaryngology surgery for laryngeal nerve reinnervation (Lee et al., 2007; Prades et al., 2015). Literature on its use in facial reanimation is scarce—non‐existent when it comes to selective transfer to individual facial nerve branches such as the MMN. Thus, as the core part of the current study, the authors aimed to examine the anatomical technical feasibility of using the ACN in selective reanimation of the MMN using cadavers. Clinically, the main goal of such transfer is to restore tone and symmetry to the lower lip in oncological patients with complete facial nerve resections, as part of the facial nerve reanimation. A clinical case is also presented, demonstrating an achievable outcome. The existing literature on the use of the ACN in facial reanimation was reviewed.

## 2 MATERIALS AND METHODS

The anatomical study was approved by the ethical institutional committee in Austria (EC number 1375/2020). The retrospective clinical case study was approved by the institutional ethical board in Sweden (Dnr 2020‐03492).

Facial dissections of 24 hemifaces in 12 fresh frozen cadavers were conducted at the Center of Anatomy, Medical University of Vienna, Austria, in October 2019. All dissections were performed under 3× loupe magnification by two senior plastic surgeons (Chieh‐Han John Tzou and Andrés Rodriguez‐Lorenzo). Eight female and four male cadavers were included (mean age 87 years [range 72–97]). Three hemifaces were excluded from the study due to pre‐existing tissue damage in the neck region.

### 2.1 Approaches and identification of ansa cervicalis

A standard triradiate type neck incision was utilized (Singh et al., 2017) The Y‐shaped incision was made with the horizontal component over the submandibular region and the vertical line just posterior to the sternocleidomastoid muscle (SCM). The usual anatomical landmarks in the area were utilized (the SCM, the internal jugular vein [IJV], the omohyoid and digastric muscles, and the hypoglossal nerve).

**FIGURE 1:**
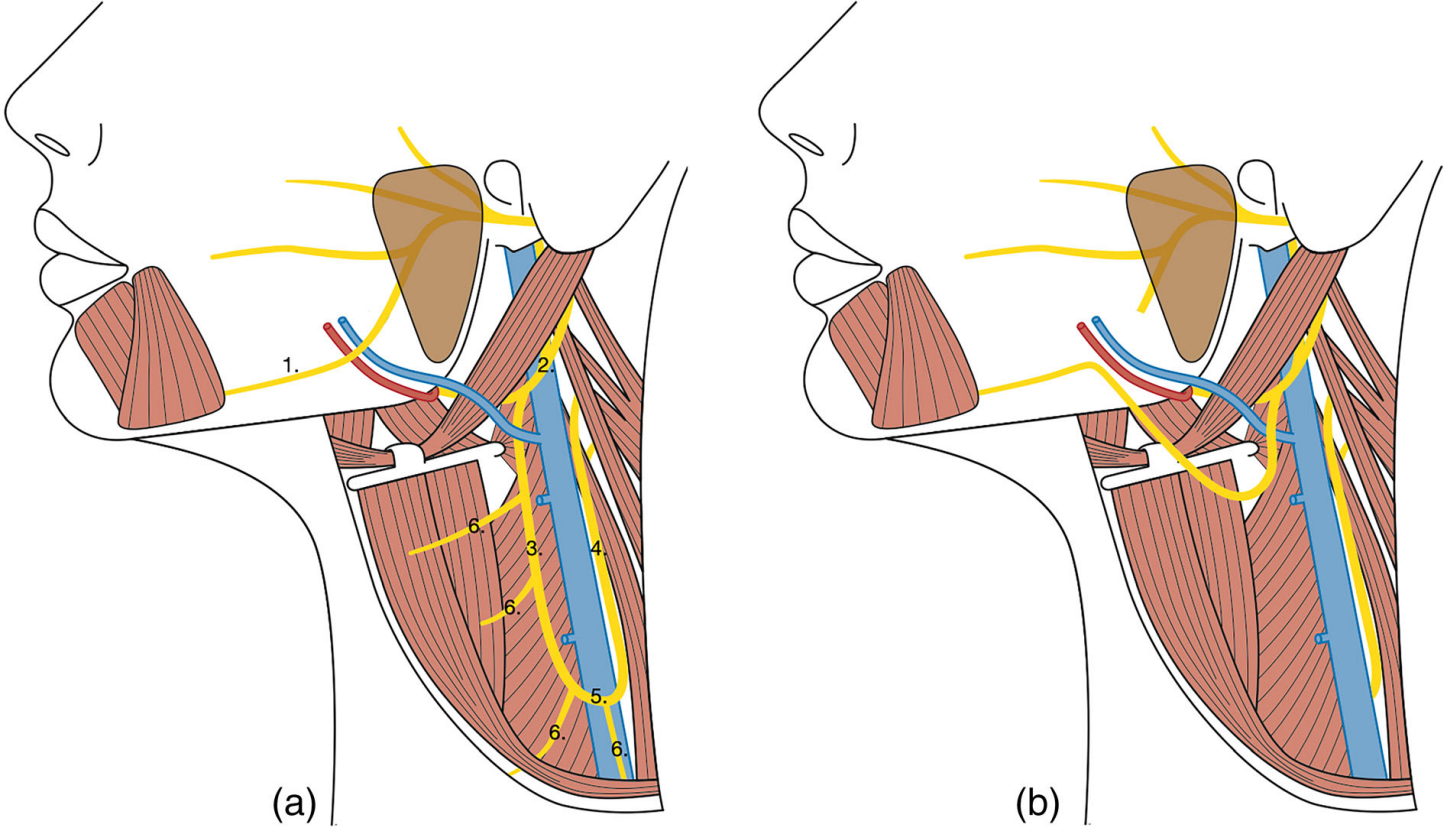
(a) Normal anatomy of the marginal mandibular nerve (MMN) and ansa cervicalis nerve (ACN). 1. MMN. 2. Hypoglossal nerve. 3. Superior root of ACN. 4. Inferior root of ACN. 5. ACN loop. 6. Branches from ACN to infrahyoid muscles. (b) Illustration of the surgical procedure—selective reinnervation of the MMN using the ACN

**FIGURE 2:**
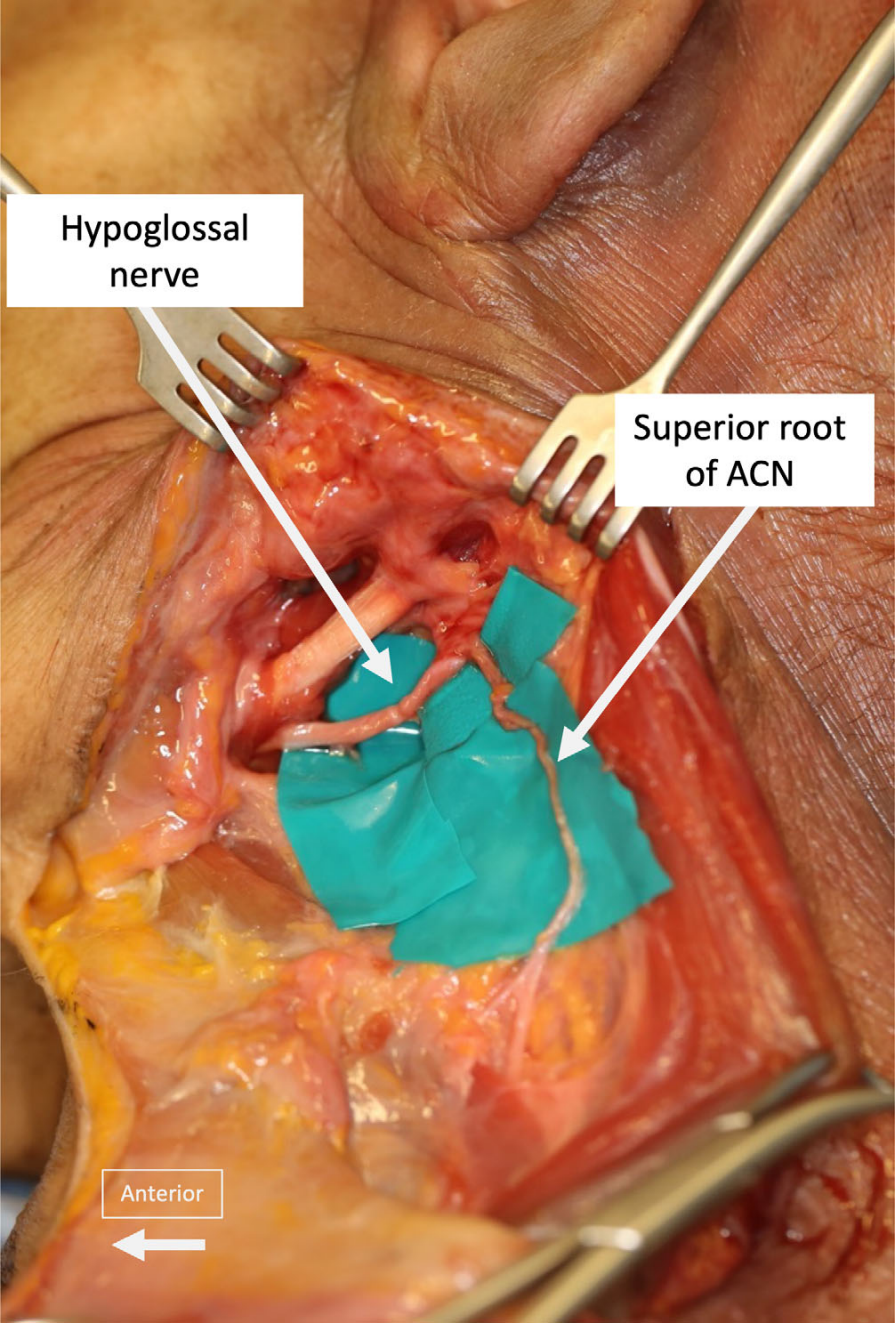
Image from dissections showing the normal ansa cervicalis nerve (ACN) anatomy, with the superior root of ACN leaving the hypoglossal nerve caudally

The superior root of the ACN was identified in one of two ways: either from the cranial direction by identifying the hypoglossal nerve deep to the posterior belly of the digastric muscle or by identifying a branch entering the omohyoid muscle and following it proximally. The course along the carotid sheath adjacent to the IJV was traced and the ACN was fully dissected from its macroscopical anatomical origin leaving the hypoglossal nerve (for ease of description, this will henceforth be called the ACN origin), and then as distally as possible until it began to loop posteriorly beyond the IJV, where it was transected (Figures 1 and 2).

### 2.2 Quantitative anatomical measurements and clinical feasibility cadaver study

First, the maximal length of the ACN that could be harvested was determined. With the cadaver's neck turned 45° to the side and a 45° backward tilt, the maximal length of harvestable ACN was measured with a caliper from the ACN origin to where it turns dorsally past the IJV (at point 5, Figure 1a). Second, the clinical feasibility of achieving tensionless coaptation between the ACN and the MMN was investigated at two anatomical locations: (1) the intersection of the MMN with the facial vessels, where most iatrogenic injuries occur (Huettner et al., 2015) and (2) at the most distal site where the MMN can be transected (at the lateral border of the depressor anguli oris). When fully dissected, the ACN was positioned at these locations to determine the reach. The outcomes were collected as either “yes” (reached target without tension) or “no” (did not). If the target was not reached, the distance (in mm) by which it fell short was measured.

### 2.3 “Short ansa” anatomical variant

**FIGURE 3:**
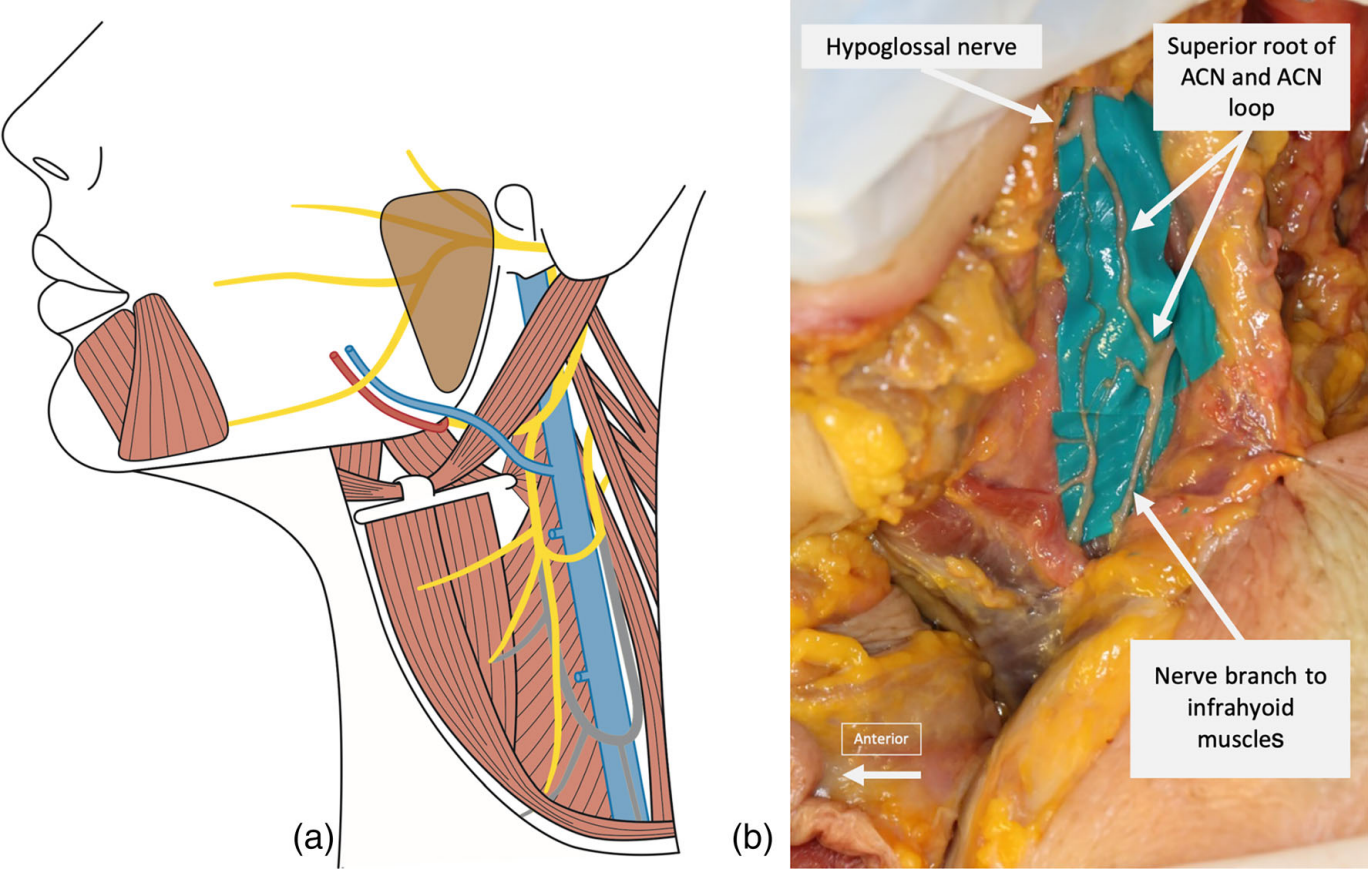
Illustration of the “short ansa” anatomical variant schematically (a) and from dissections (b). The ansa cervicalis nerve (ACN) was shorter and the ansa loop was located more superiorly than usual, compared with the normal ACN anatomy (normal anatomy colored gray in the schematic figure). Nerve branches from the ACN to the infrahyoid muscles, which can be used to extend the length of the ACN donor in these cases, can be seen leaving the ACN in an antegrade direction in both images

“Short ansa” is an anatomical variant where the inferior and superior roots of the ACN are significantly shorter than average (Chhetri & Berke, 1997) (Figure 3). In these cases, the ACN may be too short for direct coaptation to the MMN. However, the nerve length can be extended by including a nerve branch from the ACN to an infrahyoid muscle in the donor (Crumley & Izdebski, 1986; Lee et al., 2007; Olson et al., 1998; Prades et al., 2015). When one of these branches had to be used as an extension of the main nerve trunk, the potential additional length gained was measured and recorded separately. In these cases, harvestable length was recorded both with and without the extension. The prevalence of the variant was recorded, and the nerve extension workaround was explored.

## 3 RESULTS

See Table 1 for anatomical results. Fourteen hemifaces (67%) had a conventionally described ACN course. Seven hemifaces (33%) (three bilateral, one unilateral) had the anatomical variant termed “short ansa.” The mean maximal harvestable length of the ACN across all cases was 100 ± 12 mm when including the extension in the “short ansa” cases (see Section 2.3 for further information). The mean total harvestable length of the ansa cervicalis nerves with normal length (excluding all “short ansa” cases) was also 100 ± 12 mm.

**TABLE 1:** Anatomical data from the dissections

	Harvestable length of ACN (excluding the extension in the ‘short ansa’ cases)	Additional length gained in ‘short ansa’ cases by including extension	Harvestable length of ACN (including the extension in ‘short ansa’ cases)		Tension free coaptation to MMN at crossing with facial vessels	Tension free coaptation to MMN at lateral DAO border	Prevalence of ‘short ansa’ variant
Mean ± SD	79 ± 33 mm	62 ± 15 mm	100 ± 12 mm	Percentage (*n*)	100% (21/21)	100% (21/21)	33% (7/21)

*Note*: Units in millimeters.

Abbreviations: ACN, superior root of ansa cervicalis nerve; DAO, depressor anguli oris muscle; MMN, marginal mandibular nerve.

The “short ansa” variant was found to be clinically significant and relevant when the aim was to connect ACN to the MMN. In the 7 “short ansa” cases, the mean harvestable length was only 37 ± 12 mm without the extension and did not reach the MMN. A usable nerve extension was present in all these cases (100%). An additional 62 ± 15 mm length was obtained by including the nerve extension here, and the total harvestable nerve length achieved was then 99 ± 14 mm in the subgroup. In the “short ansa” cases, the possibility of tensionless coaptation was determined using the extension. This workaround enabled tensionless coaptation between the ACN and the MMN in all 21 hemifaces (100%), both at the crossing with the facial vessels and at the lateral border of the depressor anguli oris. The diameters of the ACN and the MMN grossly matched in all specimens (including the nerve branch extension in “short ansa” cases).

## 4 CASE REPORT

**FIGURE 4:**
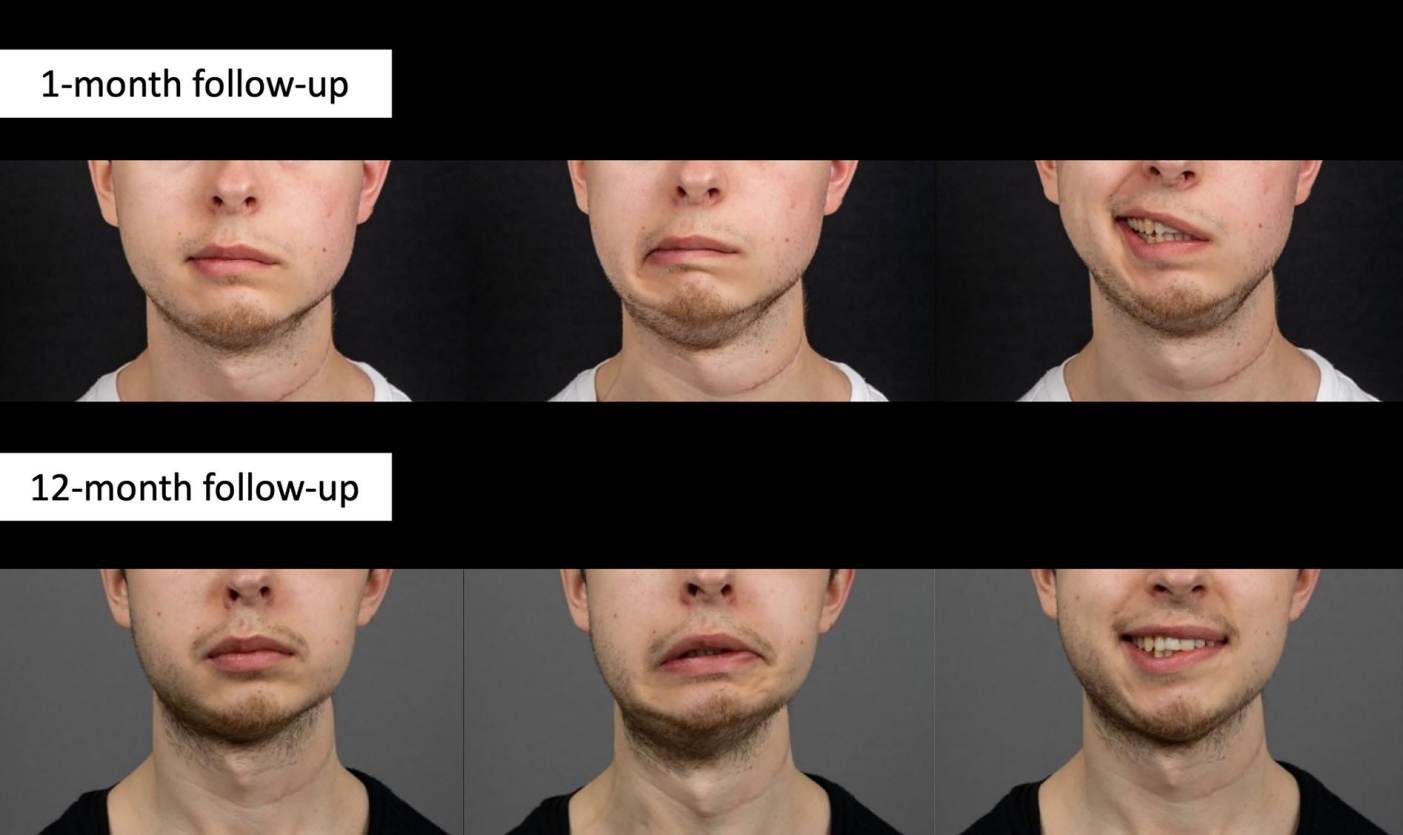
The man underwent facial nerve reconstruction with a triple facial nerve transfer: (1) the proximal facial nerve trunk was connected to the temporal and upper zygomatic branches (with a nerve graft) for forehead and eye reanimation, (2) the masseter nerve was connected to the lower zygomatic and buccal branches for smiling, (3) the marginal mandibular nerve was reinnervated with selective ansa cervicalis nerve end‐to‐end transfer for reanimation of the lower lip. Asked to show three expressions (from left: neutral, lower lip depression, smile) at follow‐up 1 month after surgery and at 12‐month follow‐up

**FIGURE 5:**
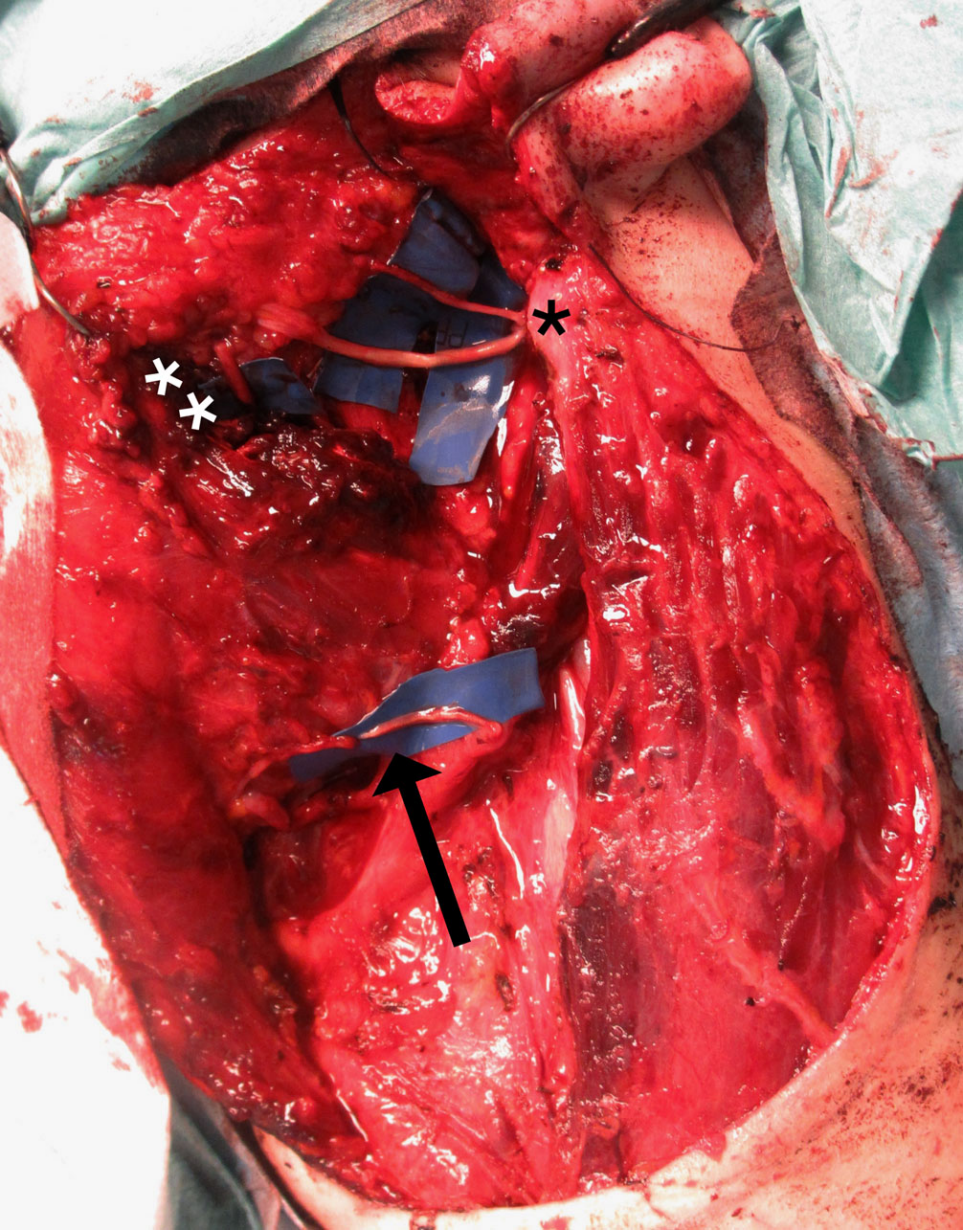
Intra‐operative image of the clinical case showing the facial nerve reconstruction. The ansa cervicalis nerve was coapted to the marginal mandibular nerve (black arrow). The proximal facial nerve trunk was connected to the temporal and upper zygomatic branches through a nerve graft (black *). The masseter nerve was connected to the lower zygomatic and buccal branches (white **)

A 28‐year‐old man presented with mammary analogue secretory carcinoma in the left parotid gland and underwent a total parotidectomy, sacrificing the facial nerve (Figure 4). The patient provided written consent for study participation and the publication of patient images and videos. Reconstruction of the facial nerve was performed using a triple facial nerve transfer, i.e., that the proximal facial nerve trunk was connected to the temporal and upper zygomatic branches (with a graft) for forehead and eye reanimation, the masseter nerve was connected to the lower zygomatic and buccal branches for smiling, and the marginal mandibular nerve was reinnervated with a local nerve transfer—the selective ansa cervicalis nerve end‐to‐end transfer—for reanimation of the lower lip (see Figure 5 for intra‐operative image). This three‐part technique is considered beneficial because of the maximization of axon supply to the targets, and the prevention of synkinesis due to the separation of donor axons. Intraoperative stimulation of the distal nerve branches was performed, which confirmed the innervation of the lower lip depressors by the MMN.

Length of follow‐up was 12 months at the time of writing. Videos and photographs of the clinical case were recorded at 1 and 12 months post‐operatively. The photographs were analyzed with photogrammetry (Emotrics; Guarin et al., 2018), where facial landmarks were marked to calculate objective differences in symmetry between facial halves. Analysis was also performed using the version of Terzis' Lower Lip Grading Scale (Terzis & Kalantarian, 2000) modified by Tzafetta et al. (2021) (lower lip function rated from 1 (poor outcome) to 5 (excellent outcome)). Both videos were graded by 25 independent observers (plastic surgery residents and specialists, medical interns, and nurses and physiotherapists from the facial palsy clinic) with 50 separate gradings in total (25 at 1 month, 25 at 12 months).

The post‐operative course was uneventful, and no donor site morbidity or clinically significant complications were seen. The subjective observer scoring of the modified Terzis' Lower Lip Grading Scale showed improvement from a mean of 1.44 (range 1–3, *n* = 25) at follow‐up 1 month after surgery to 3.24 (range 2–4, *n* = 25) at the 12‐month follow‐up. Photogrammetry demonstrated improvement in facial symmetry in all parameters during depressor activation. Asymmetry during commissure excursion improved from 18.0 to 0.6 millimeters, commissure height improved from 4.3 to 4.2, upper lip height from 3.5 to 1.2, lower lip height from 5.3 to 4.4, and smile angle from 11.4 to 8.8 (smile angle unit in degrees).

The patient underwent rigorous physiotherapy training aimed at connecting lower lip movement to the previous ACN function of swallowing. Clinically, apart from improved tone and symmetry which was the main goal of the selective procedure, this also resulted in active contraction of the lower lip depressors on the previously paralyzed side, including clinically verified contraction of the depressor labii inferioris muscle (Figure 4, Supplemental video). Independent lower lip activation was seen without swallowing, indicating that lower lip movement was released from original ansa cervicalis function. Furthermore, an effortless smile was achieved without biting, indicating that the upper part of the smile was disconnected from the masseter nerve. This, together with the fact that the cervical branch was severed in the tumor resection, indicates that MMN function was restored solely through the ACN donor. Slight asymmetry was still present with a full denture smile, where botox at the contralateral side may further enhance cosmetic results. An interesting detail is that lower lip atrophy has not occurred on the affected side in this case, and the lip volume is maintained, probably due to the tone provided by the ACN transfer. Some patients require filler procedures or injections because of this atrophy, which might be avoided when utilizing the selective ACN transfer.

## 5 DISCUSSION

In this study, the authors examined the use of the superior root of the ACN for selective reinnervation of the MMN in cadavers. The goal was to provide a new nerve donor to the lower lip for restoration of tone and symmetry in oncological facial paralysis patients undergoing a triple facial nerve transfer (see Section 4 for definition of the triple transfer). The study contributes novel information in the following four areas: (1) new anatomical details are presented for utilization of ACN as a selective donor nerve in the face, (2) the clinical relevance of the anatomical variant “short ansa” is described when connecting ACN to the facial nerve, (3) a case report undergoing ACN to facial nerve transfer is analyzed using both objective and subjective methods, and (4) a summary of the literature provides a potential explanation for why the ACN has previously been under‐utilized by plastic surgeons.

The ACN to facial nerve transfer has been reported previously with varying techniques and results, but the selective transfer of ACN to a single facial nerve branch has not been studied. A literature search was conducted (PubMed) with search terms “ansa cervicalis” OR “ansa hypoglossi” OR “descendens hypoglossi” AND “facial” OR “marginal mandibular.” Articles on facial nerve reinnervation using ACN and their references were examined and included if they reported on outcome data. The literature search yielded only seven published studies from 1979 through to today (Arndt et al., 2004; Conley & Baker, 1979; Gidley et al., 2010; Leong & Lesser, 2013; Liang et al., 2015; Schipper et al., 2005; Steiner et al., 1997). All studies used the ACN as the sole donor for the entire facial nerve stump (i.e., for all five branches). Four seemed to show that the ACN could adequately power the entire facial nerve. For example, in the largest series utilizing the ACN for reinnervation of the main facial nerve trunk, 85% achieved “excellent” or “sufficient” restoration of perioral resting tone (Steiner et al., 1997). Sixty‐six percent had “excellent” active perioral motility and 15% had “unsatisfactory” motility. Active lid closure was possible in 80% of patients, but unsatisfactory in 28% of these. Two articles reported House Brackmann grades II–III in 75% (Schipper et al., 2005) and 91% (Arndt et al., 2004) of cases, respectively; one reported House Brackmann grades II–IV in 91.7% (Liang et al., 2015). An unpublished retrospective preprint compared ACN‐facial nerve trunk anastomosis to end‐to‐side hypoglossal‐facial anastomosis (Zhou et al., 2022). Outcomes were similar between the groups, with “favorable” results achieved in 68.8% and 70% of case, respectively (it should be noted that only patients with large ACN diameters were selected for ACN‐facial anastomosis).

Despite these promising results, it seems unlikely that the ACN can provide enough axons for consistent and dependable reinnervation of the entire facial nerve across the patient population. Associated synkinesis, which few of the above articles discuss, is likely hard to avoid using this method. Conley first reported that they “uniformly required additional supplementary techniques to support the face” when reinnervating the trunk (Conley & Baker, 1979). Gidley et al. reported “very poor” results, with their one patient having no effect from the ACN‐facial transfer (Gidley et al., 2010). The best results achieved by Leong et al. were House Brackmann grade IV (Leong & Lesser, 2013). Doubts have been raised regarding if the ACN can provide an adequate axon count in this context (Hammerschlag, 1999; Manni et al., 2001; Meybodi et al., 2020; Vacher & Caix, 2004; Vacher & Dauge, 2004), in part because the descending superior root of the ACN also contains ascending nerve fibers from the inferior root, hypothetically leading to poor nerve signaling (Banneheka, 2008; Vacher & Caix, 2004). These doubts were all raised in the context of reinnervating the relatively large targets of the main facial nerve trunk and the hypoglossal nerve, with axon counts around 6000–7000 (Asaoka et al., 1999; Captier et al., 2005; Engelmann et al., 2020; Hembd et al., 2017; Kondo et al., 2011) and 9500 (Asaoka et al., 1999; Mackinnon & Dellon, 1995), respectively. A substantial size mismatch exists between the ACN and the main extratemporal facial nerve trunk, with the ACN providing only 20% of the fascicular surface area of the trunk (Vacher & Dauge, 2004) and with a diameter jump of 1:3 between the nerves (Schipper et al., 2005). While this mismatch can explain the unpredictable results described above when innervating the full facial nerve with the ACN, and thus shed light on the hesitancy of plastic surgeons to adopt the technique, its use for reinnervating a single branch of the facial nerve—such as the MMN—should be consistently reliable. The MMN has an axon count of only 1603 ± 849 (Mandlik et al., 2019), and the nerve diameters of the ACN and MMN match, both in the literature (Chhetri & Berke, 1997; Loukas et al., 2007; Mandlik et al., 2019; Prades et al., 2015) and in the current study. The fact that the ACN has been used in the field of otorhinolaryngology for a century (Frazier, 1924) and remains the donor nerve of choice for the size‐wise similar laryngeal nerve (Ayyoubian & Koruji, 2011; Chhetri & Berke, 1997; Chhetri & Blumin, 2012; Crumley & Izdebski, 1986; Lee et al., 2007; Loukas et al., 2007; Olson et al., 1998; Prades et al., 2015; Wang et al., 2011) further strengthens its potential as a reliable selective donor nerve for the MMN. A further advantage of using the ACN as a selective donor for MMN in a triple facial nerve transfer, rather than as the sole donor for the full facial nerve, is the reduced risk of synkinesis.

### 5.1 The versatility of the ACN as a donor nerve

The cadaver study showed that the ACN could be identified using standard neck dissection landmarks in all cases. The ACN had a mean maximal harvestable length of 100 ± 12 mm (including the extension in “short ansa” cases). This allowed tensionless coaptation to the MMN in all hemifaces examined, even at the most distal possible transection site. During the study, the potential of the ACN to reach other branches of the facial nerve at common transection sites was also examined as a separate subject of interest. Tensionless adaptation to the zygomatic and buccal branches of the facial nerve was found to be possible in all specimens. This may add to the versatility of the ACN, for example in cases where the masseteric nerve is not available.

### 5.2 “Short ansa” anatomical variant

The anatomical variant “short ansa” was present in 33% of cadaver hemifaces (*n* = 7), a prevalence consistent with previously reported findings (Chhetri & Berke, 1997). Here, the ACN was significantly shorter than usual, with a mean length of only 37 ± 12 mm, not allowing tensionless coaptation to the MMN. To overcome this issue, an infrahyoid muscle nerve branch was included in the final nerve donor as an extension. This extension has earlier been used in otolaryngology surgery, but has, to the authors knowledge, never been explored for application in plastic surgery before the current study. Our results show that donor length in “short ansa” cases can be increased to 99 ± 14 mm by including the extension, very close to the 100 ± 12 mm of the normal length ACN. This allowed for tensionless coaptation to the full length of the MMN in all hemifaces dissected (Table 1). Here, the common nerve branch to the sternothyroid and sternohyoid muscle was used primarily, but other branches are also available (Crumley & Izdebski, 1986; Lee et al., 2007; Olson et al., 1998; Prades et al., 2015). The ACN diameter, which matches the diameter of the MMN, seems to be preserved in these branches (Chhetri & Berke, 1997; Loukas et al., 2007; Mandlik et al., 2019; Prades et al., 2015). No “short ansa” variant was encountered in the clinical case.

### 5.3 Proposed indication for selective ACN‐MMN transfer

The authors propose the ACN as a selective donor nerve for the MMN in oncological patients with complete facial nerve resections requiring neck dissection, with the goal of restoring tone and symmetry to the lower lip. The transfer is suggested as part of a triple facial nerve reanimation (defined above), to maximize axon supply to the face and avoid synkinesis. Nerve transfer is an attractive treatment option here, not only because the single coaptation and use of a vascularized nerve are important factors in an area designated for radiation therapy (Klein et al., 2019), but also because of the shorter reinnervation time following a more distal direct innervation, which can be crucial in patients with potentially poor prognosis. In these cases, the ACN is a clear choice for direct MMN reconstruction because of the easy nerve access in the already generous surgical field, together with the aforementioned benefits. While the main goal of the ACN‐MMN transfer in the intended patient demographic is restoration of tone and symmetry to the lower lip, return of active contraction can also be achieved with rigorous physiotherapy training (Figure 4 and Supplemental video).

### 5.4 Potential contraindications and limitations

While the morbidity of ACN transection is reported to be nearly negligible (Ayyoubian & Koruji, 2011; Chhetri & Berke, 1997; Liguoro et al., 1992; Loukas et al., 2007; Prades et al., 2015; Quadros et al., 2013; Souza & Ray, 2010; Steiner et al., 1997; Vollala et al., 2005), hoarseness, throat tightness, swallowing difficulties, and changes in voice quality are potential sequalae to be aware of (Mnatsakanian & al Khalili, 2022). Paralysis of the infrahyoid muscles in professional voice users may result in phonation problems and loss of pitch but does not lead to voice problems in normal speech (Ayyoubian & Koruji, 2011). These disturbances may need to be considered in a select group of patients.

The usual limitations of translating results from cadaver studies into a clinical context apply to this study. The diameters of the nerves were not measured, with only gross comparisons done. Some bias might exist in the Terzis' analysis of the case report, as observers were shown the two videos in chronological, and not randomized, order. No information on if the images were pre‐ or post‐op was provided however, and observers were asked to rate the videos without further background information.

## 6 CONCLUSIONS

Selective ACN‐MMN nerve transfer is anatomically feasible and should be considered as part of a triple facial nerve transfer in oncological ablative procedures in patients requiring neck dissection. The goal of the transfer is to provide improved tone and symmetry to the lower lip, but active movement can also be achieved. A novel strategy for when encountering the “short ansa” anatomical variant is proposed: the ACN length can be extended by including an infrahyoid muscle nerve branch in the donor. The clinical case showed good outcomes and anatomy that corresponded to the findings in the cadaver study. The literature seems to support the relatively low morbidity of the donor site. Clinical studies are needed to assess the reliability of the selective transfer.

## FUNDING INFORMATION

This research was in part financed with the Swedish Research Council's funding for clinical research in medicine (ALF).

## CONFLICT OF INTEREST

The authors declare no conflict of interest.

## Supporting information


**Data S1.** Supporting information

## Data Availability

The data that support the findings of this study are available from the corresponding author upon reasonable request.
